# The compound TB47 is highly bactericidal against *Mycobacterium ulcerans* in a Buruli ulcer mouse model

**DOI:** 10.1038/s41467-019-08464-y

**Published:** 2019-01-31

**Authors:** Yang Liu, Yamin Gao, Jianxiong Liu, Yaoju Tan, Zhiyong Liu, Chiranjibi Chhotaray, Huofeng Jiang, Zhili Lu, Gift Chiwala, Shuai Wang, Gaelle Makafe, Md Mahmudul Islam, H. M. Adnan Hameed, Xingshan Cai, Changwei Wang, Xinjie Li, Shouyong Tan, Tianyu Zhang

**Affiliations:** 1 State Key Laboratory of Respiratory Disease, Guangzhou Regenerative Medicine and Health Guangdong Laboratory (GRMH-GDL), Guangzhou Institutes of Biomedicine and Health (GIBH), Chinese Academy of Sciences (CAS), Guangzhou, 510530 China; 2 Institute of Physical Science and Information Technology, Anhui University, Hefei, 230601 China; 3 University of Chinese Academy of Sciences (UCAS), Beijing, 100049 China; 4 State Key Laboratory of Respiratory Disease, Department of Clinical Laboratory, Guangzhou Chest Hospital, Guangzhou, 510095 China; 5 School of Life Sciences, University of Science and Technology of China, Hefei, 230027 China

## Abstract

Buruli ulcer (BU) is an emerging infectious disease that causes disfiguring skin ulcers. The causative agent, *Mycobacterium ulcerans*, secretes toxin called mycolactone that triggers inflammation and immunopathology. Existing treatments are lengthy and consist of drugs developed for tuberculosis. Here, we report that a pyrazolo[1,5-a]pyridine-3-carboxamide, TB47, is highly bactericidal against *M. ulcerans* both in vitro and in vivo. In the validated mouse model of BU, TB47 alone reduces *M. ulcerans* burden in mouse footpads by more than 2.5 log_10_ CFU compared to the standard BU treatment regimen recommended by the WHO. We show that mutations of ubiquinol-cytochrome C reductase cytochrome subunit B confer resistance to TB47 and the dissimilarity of CydABs from different mycobacteria may account for their differences in susceptibility to TB47. TB47 is highly potent against *M. ulcerans* and possesses desirable pharmacological attributes and low toxicity that warrant further assessment of this agent for treatment of BU.

## Abstract (web summary)

Combination therapy for Buruli ulcer (BU) is suboptimal. Here, Liu et al. show that the candidate drug TB47 has potent bactericidal activity against *Mycobacterium ulcerans* in vitro and in a mouse model, which underscores its potential for shortening the course of BU and treating other mycobacterial diseases.

## Introduction

Buruli ulcer (BU)^1–3^, caused by *Mycobacterium ulcerans*, is the third prevalent mycobacterial disease, after tuberculosis (TB) and leprosy. The World Health Organization (WHO) recommends a combination of rifampin and streptomycin for 2 months for treatment of BU following evaluation of this regimen in a murine model of BU^3^. Although this treatment is suboptimal as streptomycin needs to be injected daily for at least 2 months and may result in hearing loss^4,5^, surgery that was previously the only possible therapeutic intervention became only adjuvant treatment for serious case. All potential oral regimens^4,5^ repurposed from TB treatment arsenal are not obviously more potent against *M. ulcerans* in vivo.

It takes ~3 months for *M. ulcerans* to form a visible colony on agar supplemented with enriched media specific for mycobacterial growth. Traditional methods that depend on enumerating colony-forming units of bacteria to evaluate efficacy of a drug would significantly prolong the duration of preclinical studies required for development of anti-BU treatment^6,7^. To address this concern, an autoluminescent reporter *M. ulcerans* strain (AlMu), which spontaneously emits light without the addition of exogenous substrate was created using the *luxCDABE* operon from *Photorhabdus luminescens* and used for anti-BU drug discovery including for in vivo efficacy evaluation^6^. That study assessed activities of a panel of drugs with drugs with different mechanisms of actions and drug combinations and demonstrated that relative light units (RLU) counts correlated well with colony-forming unit (CFU) of *M. ulcerans*^6^. Use of this technology enabled rapid (requiring only 3 s) and serial real-time monitoring of *M. ulcerans* in samples in vitro and in the same batch of small live animals for evaluation of drug activity. Therefore, this approach can drastically reduce the time, effort, animals, and resources necessary for in vitro and in vivo assessment that require monitoring of *M. ulcerans* growth. Here, we used AlMu to evaluate our small compounds library to discover agents to develop new anti-BU treatment.

We recently reported pyrazolo[1,5-a]pyridine-3-carboxamides exhibit activity against *Mycobacterium tuberculosis*^8^. The lead compound of this class, TB47, is now being developed as an anti-TB drug (http://www.newtbdrugs.org/pipeline/discovery). Its mechanism of action is unknown and agents with this pharmacophore have not been evaluated against non-tuberculous mycobacteria. In this study, we found TB47 to be highly potent against *M. ulcerans*^9^. We also identified the target inhibited and the mechanism by which resistance to TB47 may arise.

## Results

### Bactericidal activity against *M. ulcerans* in vitro

**Fig. 1:**
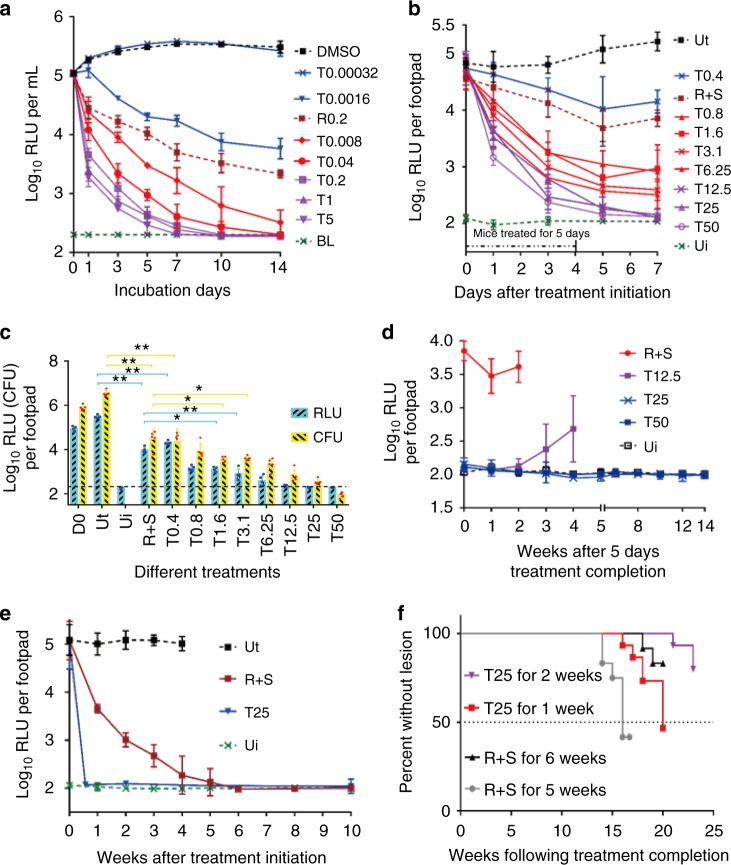
Activity of TB47 (T) against *M. ulcerans*. **a** Time-killing curves of TB47 against AlMu in liquid culture. Concentrations (μg mL^−1^). Data are expressed as mean ± SD from three independent biological repeats. The experiment was performed in triplicate (three independent experiments) and the representative results are shown. **b**–**d** Mice were treated from 12 days post infection of AlMu (ALI = 1.09 ± 0.28). Data are expressed as mean ± SD of five samples. **b** RLUs detected from left hind footpads of the same batch of live mice treated for 5 days and stopped for 3 days. Statistical analysis was performed using unpaired Student’s *t*-test. **c** RLUs and CFUs of footpad tissue suspension at day 0 and day 7. The experiments for **b**, **c** were performed in triplicate (three independent experiments) and the representative results are shown. The correlation coefficients (*R*^2^) of RLU and CFU for the in vivo experiments were 0.966 (including the positive) and 0.898 (TB47 treated groups only), respectively. Correlation analysis was performed using Pearson’s correlation test. The linearity of the relationship of RLU and CFU for the footpad suspension is valid when the CFUs are from 2.5 to 6.2 log_10_ CFU mL^−1^. Statistical analysis was performed using unpaired Student’s *t-*test, *P < 0.001; **P < 0.0001. **d** Footpad RLUs detected after completion of treatment for monitoring relapse. **e** Kinetic curves of RLUs from the footpads of live mice treated with different regimens. Data are expressed as mean ± SD of five samples. **f** Time to footpad swelling after completion of antibiotic treatment. Time to footpad swelling in mice treated with either rifampin + streptomycin for 5 weeks (gray circles) or 6 weeks (black triangles), or TB47 for 1 week (red square) or for 2 weeks (purple triangle). Statistical differences were determined by Log-rank (Mantel-Cox) test with 15 mice in each group. T, TB47; BL, base line; Ui, uninfected (for RLU detection, Ui is the base line); Ut, untreated; R, rifampin, 25; S, streptomycin, 150; Dosage (mg kg^−1^). The dotted green lines indicated the base line (the limit of detection). Data are expressed as mean ± SD. ^*^P < 0.001; ^**^P < 0.0001

We determined the minimum inhibitory concentrations (MICs) of TB47 against multiple non-tuberculous mycobacteria and a range of clinically important bacteria using standard broth dilution assay as per the Clinical and Laboratory Standard Institute Guidelines (Supplementary Table 1). While its MICs (µg mL^−1^) were > 128 for most of the clinical strains, the lowest MICs (0.0016) were observed for the two different *M. ulcerans* isolates. These data indicated that TB47 is specific to and highly potent against *M. ulcerans*. Next, we used the standard time kill assay to determine whether TB47 was bacteriostatic or bactericidal against *M. ulcerans*. In this assay, TB47 demonstrated bactericidal activity at concentration ≥ 0.0016 µg mL^−1^ (Fig. 1a). The bactericidal activity of TB47 at 0.008 µg mL^−1^ or higher was more potent than that exhibited by 0.2 µg mL^−1^ rifampin, a drug currently used to treat BU. The bactericidal activity of TB47 was also verified by enumerating CFU (Supplementary Fig. 1). Its minimum bactericidal concentration (MBC) against *M. ulcerans* is 0.0064 µg mL^−1^.

### In vivo bactericidal activity of TB47 against *M. ulcerans*

**Fig. 2:**
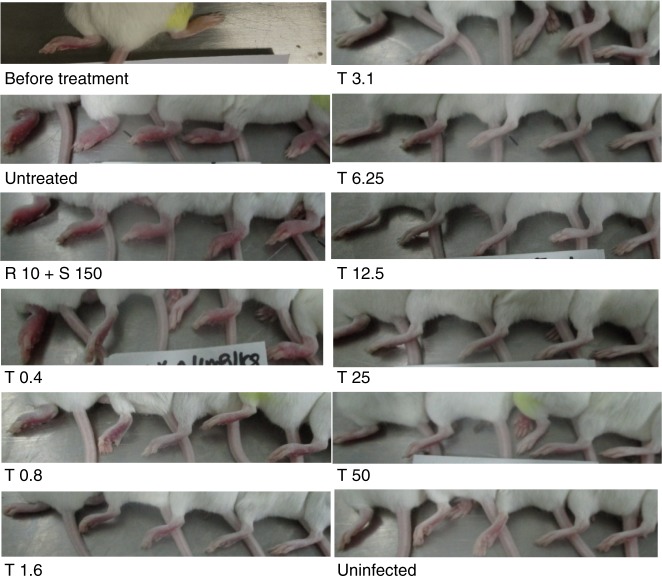
Photographs showing the swelling degrees of the left hind mouse footpads at 0 and day 7. The ALI is 3 ± 0, 2.8 ± 0.27, and 1.8 ± 1.25, for the untreated, rifampin + streptomycin and T0.4, respectively. Only one obvious swelling footpad (swelling index about 2) was observed in the T0.8 mg kg^−1^ group (the left one) and one questionable swelling footpad in the T0.8 mg kg^−1^ group (the middle one) and no any swelling footpad (ALI = 0) was observed in any groups treated with *T* ≥ 3.1 mg kg^−1^. T, TB47; R, rifampin; S, streptomycin. Dosage, mg kg^−1^

We evaluated activity of TB47 in a validated mouse model of BU^4,6,7^ using AlMu generated from *M. ulcerans* 1059^6^. TB47 exhibited bactericidal activity even at ≥ 0.4 mg kg^−1^, the lowest among doses we evaluated in the mouse model of BU. TB47 alone at 0.8 mg kg^−1^ exhibited efficacy superior to the current WHO recommended standard regimen to treat Buruli ulcer which includes a combination of 10 rifampin and 150 streptomycin (doses mg kg^−1^) in the live mouse model (Fig. 1b, *P* < 0.001). At doses ≥ 12.5 mg kg^−1^, the bioluminescence detected 3 days after treatment completion (at day 7) in live mice was indifferent from the background reading (Fig. 1b, *P* > 0.05). The classical disease presentation in the mouse model of BU includes swelling, redness and tenderness of the footpads (the site of *M. ulcerans* injection). In mice treated with ≥ 3.1 mg kg^−1^ TB47, at day 7, we observed no swelling or redness in the footpads of mice with average lesion index^10^ (ALI) = 0, while those treated with the standard regimen containing rifampin and streptomycin exhibited ALI = 3 (Fig. 2). Alternatively, we used bioluminescence and CFUs to quantify *M. ulcerans* burden in the footpad tissue at day 7. This assessment also showed > 2 log_10_ reduction in RLU in the footpad tissue of mice treated with ≥ 3.1 mg kg^−1^ TB47. The minimal bactericidal dose (MBD) was 1.6 mg kg^−1^ and at doses ≥ 12.5 mg kg^−1^, RLUs were reduced to the background level. *M. ulcerans* burden (CFU per footpad) in mice treated with 50 mg kg^−1^ of TB47 was ~3.9 log_10_ lower than that at day 0 and ~2.5 log_10_ lower than in mice treated with the standard regimen containing rifampin and streptomycin (Fig. 1c).

### Preliminary sterilizing activity

For mice treated with ≥ 25 mg kg^−1^ of TB47, RLU could not be detected at significant levels even up to 14 weeks post completion of treatment (Fig. 1d). However, *M. ulcerans* infection relapsed in 4 to 6 out of 10 mice at 20 to 24 weeks (ALI > 1, Supplementary Fig. 2). In a separate experiment, mice treated with 25 mg kg^−1^ TB47 for 1 or 2 weeks were compared with mice treated with the standard regimen containing rifampin and streptomycin for 5 or 6 weeks. RLUs from footpads of live mice treated with the standard regimen for 4 weeks were still above the background reading, while the RLUs from footpads of live mice treated with TB47 for 5 days reached the background noise levels (Fig. 1e). In comparison to the standard regimen for 5 weeks, 25 mg kg^−1^ TB47 for 1 week significantly extended the time-to-replase (*P* < 0.01; Fig. 1f). Also, longer duration of treatment of TB47 further prolonged time-to-relapse.

### Resistance to TB47 due to QcrB

**Fig. 3:**
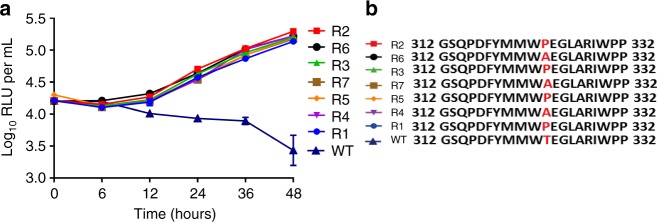
Drug susceptibility testing and sequence analysis of resistant mutants. **a** Time-killing cures of TB47 (1 μg mL^−1^) against autoluminescent *M. marinum* resistant mutants (R1 to R7) and the parent strain in liquid culture. Data are expressed as mean ± SD from three independent biological repeats. The experiment was performed in triplicate (three independent experiments) and the representative results are shown. **b** Results of polymorphism in QcrB conferring resistance to TB47. WT, wild-type

To explore its mechanism of action, *Mycobacterium marinum*, a close relative of *M. ulcerans* with > 98% genome sequence identity^2^, was used at first as it takes only about 2 weeks for *M. marinum* to form a visible colony on the agar plate. We were able to select *M. marinum* spontaneous mutants in the presence of TB47 and confirmed that the isolates stably exhibited resistance to TB47 (Fig. 3a). The isolates exhibited a consistent increase in MICs for TB47 but remained susceptible to the control drug rifampin (Table 1). Similarly, nine TB47-resistant *M. ulcerans* colonies were isolated very recently only on plates containing 0.02 μg mL^−1^ TB47 but none on plates containing ≥ 0.05 μg mL^−1^ TB47, although we attempted > 10 screens at  ≥ 0.05 μg mL^−1^ of TB47. Based on three independent mutant-selection attempts using *M. marinum* and *M. ulcerans*, we determined spontaneous resistance mutation rates against TB47 to be 0.45 × 10^−8^ for *M. marinum* at 1 μg mL^−1^ and 0.83 × 10^−9^ for *M. ulcerans* at 0.02 μg mL^−1^.

**Table 1:** Susceptibilities of different *M. marinum*/*M. ulcerans* strains to TB47 and rifampin in liquid using bioluminescence

Mycobacteria	Gene: *qcrB* from *M. marinum*	MIC_lux_s (μg mL^−1^)	
*M. marinum* strains		TB47	RIF
**Wild-type (Autoluminescent strain)**	Wild-type	0.001–0.008	0.25–1.0
**Type 1 mutant**	ACC to CCC (Thr323Pro)	> 128	0.25–1.0
**Type 2 mutant**	ACC to GCC (Thr323Ala)	> 128	0.25–1.0
**Wild-type:: vector**	Wild-type containing an empty plasmid	0.001–0.008	0.25–1.0
**Wild-type::** ***qcrB*** ^**WT**^	Over-expression of wild-type *qcrB*	32–64	0.25–1.0
**Wild-type::** ***qcrB*** ^**THR323PRO**^	Over-expression of *qcrB*^Thr323Pro^	> 128	0.25–1.0
**Wild-type::** ***qcrB*** ^**THR323ALA**^	Over-expression of *qcrB*^Thr323Ala^	> 128	0.5–2.0
***M. ulcerans*** **strains**		**TB47**	**RIF**
**Wild-type (AlMu 1059)**	Wild-type	0.0016	0.06–0.25
**Wild-type:: vector**	Wild-type containing an empty plasmid	0.0016–0.0031	0.06–0.25
**Wild-type::** ***qcrB*** ^**WT**^	Over-expression of wild-type *qcrB*	0.05–0.10	0.06–0.25
**Wild-type::** ***qcrB*** ^**THR323PRO**^	Over-expression of *qcrB*^Thr323Pro^	0.10 to 0.40	0.06–0.25
**Wild-type::** ***qcrB*** ^**THR323ALA**^	Over-expression of *qcrB*^Thr323Ala^	0.10 to 0.40	0.125–0.5

MIC minimum inhibitory concentration

We sequenced the genomes of seven independent TB47-resistant *M. marinum* isolates and the parent strain. We identified single-nucleotide polymorphisms (SNPs) at the same codon in all resistant isolates ACC→GCC or CCC resulting in Thr323Ala or Thr323Pro substitution in QcrB (ubiquinol-cytochrome C reductase cytochrome subunit B) (Table 1). We amplified this locus in an additional 20 independent spontaneous *M. marinum* mutants, sequenced and found mutations only in the same codon that resulted in Thr323Ala or Thr323Pro substitutions (Table 1 and Fig. 3b). Alignment of amino acid sequences of QcrBs from *M. ulcerans* and *M. marinum* revealed 100% identity (Supplementary Fig. 3a). Similarly, we sequenced *qcrB* gene in 9 independent spontaneous *M. ulcerans* mutants. Eight out of nine mutants harbored the SNP ACC→ GCC resulting in Thr323Ala while the remaining isolate harbored SNP ACC→ ATC resulting in Thr323Ile. To verify if such substitutions truly lead to resistance to TB47, we overexpressed wild-type and mutated *qcrB* genes in *M. marinum* and *M. ulcerans*, and observed an increase in MICs of TB47 (Table 1). Based on these data, we conclude that mutations of QcrB Thr323 confer *M. marinum* and *M. ulcerans* resistance to TB47.

### Two terminal oxidases in electron transport chain

**Fig. 4:**
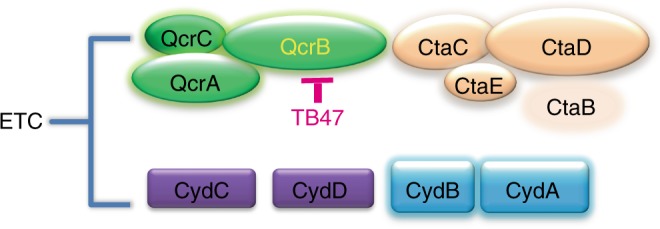
Two terminal oxidases in the electron transport chain of mycobacteria. Mutations in QcrB in the cytochrome bc1:aa3 confer resistance to TB47

**Fig. 5:**
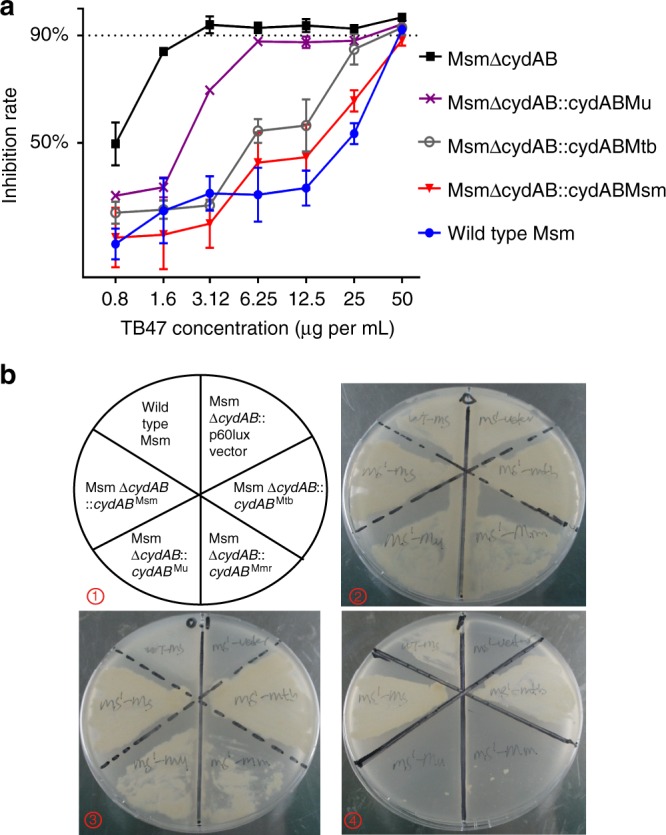
Effect on the sensitivity to TB47 during the over-expression of CydABs in *M. smegmatis*. **a** The inhibition rates against *M. smegmatis* mc^2^ 155 and derivative strains. Bacteria treated with different concentrations of TB47 and fluorescence units (FU) were recorded 4 h post-alamar blue addition. The inhibition rate was calculated as FU_treatment_ /FU_untreated_ × 100% and recorded for each strain per treatment. Data are expressed as mean ± SD from three independent repeats. **b** Inhibition growth of different *M. smegmatis* strains by TB47 at different concentrations. ① The plate lay-out is shown (*M. smegmatis* containing the construct detailed). ② to ④ showing the plates containing TB47 at 0, 0.1, and 1 (μg mL^−1^) incubated at 37 °C for 36 h. Msm, *M. smegmatis*; Mu, *M. ulcerans*; Mtb, *M. tuberculosis*; Mmr, *M. marinum*

The electron transport chain of mycobacteria usually contains two terminal oxidases, the cytochrome bc1:aa3 containing QcrCAB and the cytochrome bd oxidase (Cyt-bds) containing CydAB (Fig. 4). Inhibition of the bc1:aa3 complex is only bacteriostatic because the alternate Cyt-bds is capable of maintaining a membrane potential and menaquinol oxidation and is therefore sufficient to maintain respiration to protect *M. tuberculosis* from death^11^. So if TB47 inhibits QcrB, it should be more powerful against the strain lacking of functional Cyt-bds. The *cydAB* genes were deleted in the model organism *M. smegmatis* to generate a *ΔcydAB* strain (Msm Δ*cydAB*). The synthetic lethal interaction between the Cyt-bc1:aa3 and the Cyt-bds was evaluated by treating the MsmΔ*cydAB* with TB47. A synthetic lethal interaction is a well-described phenomenon where simultaneous inactivation of two genes that confer an essential activity results in cell death but inactivation of either gene alone does not affect cell viability. Similar to a synthetic lethality described in *M. tuberculosis*^11^, deletion of *cydAB* in *M. smegmatis* did not impact significantly on bacterial growth (Supplementary Fig. 4) but sharply increased the inhibitory potency of TB47 (Fig. 5a) as its MIC was reduced from 50 to 3.12 μg mL^−1^.

### Activities of Cyt-bds from different mycobacteria

The amino acid sequences of QcrB, CydA, and CydB from different mycobacteria were aligned (Supplementary Fig. 3a, b, c). The QcrB as shown before^12^, is highly conserved among mycobacteria, but the similarity of CydA/B between *M. tuberculosis* and that of *M. smegmatis* or *M. ulcerans* was low. It is interesting that *M. leprae* genome altogether lacks genes that encode CydA/B. This led us to hypothesize that the Cyt-bds from different mycobacteria may not have identical activities, which potentially explained the distinct susceptibilities of mycobacteria to TB47.

To verify this hypothesis, we created a series of recombinant strains by introducing *cydAB* genes from different mycobacteria into Msm*ΔcydAB*. The MICs of TB47 against Msm*ΔcydAB*::*cydAB*^*Msm*^, Msm*ΔcydAB*::*cydAB*^*Mtb*^, Msm*ΔcydAB*::*cydAB*^*Mu*^, and Msm*ΔcydAB* were 50, 37.5, 6.5, and 3.2 μg mL^−1^, respectively (Fig. 5a), while MICs of isoniazid were 4 μg mL^−1^ for all four strains. In the presence of TB47, these strains exhibited different in vitro growth profiles (Fig. 5b). *M. ulcerans cydAB* could not sufficiently complement the function of *M. smegmatis* Cyt-bds. This indicated that intrinsic activity of Cyt-bds of *M. ulcerans* could be too weak, which may explain well why TB47 had surprising bactericidal activity against *M. ulcerans*.

### Pharmacological attributes and low toxicity of TB47

There is no obvious toxicity of TB47 observed until now. TB47 did not display obvious cytotoxicity with IC_50_ of >100 µM for VERO cell and 50 µM for THP-1 cell. TB47 did not inhibit hERG (IC_50 _> 30 µM), suggesting a low risk for cardiotoxicity. A single oral administration of 2000 mg kg^−1^ of TB47 or oral 200 mg kg^−1^ daily for 4 weeks, the highest dosage we tested, was well tolerated by mice. No genotoxicity or obvious CYP inhibition was associated with this compound, and its metabolic stability in different animal species and permeability are high (Supplementary Table 2). Furthermore, in a long-term administration study, TB47 was well tolerated without any meaningful clinical signs of toxicity when administered daily at a dose of 10, 30, or 100 mg kg^−1^ body weight orally for 30 days using both male and female rats. These data demonstrate that TB47 is well tolerated even during prolonged use.

**Fig. 6:**
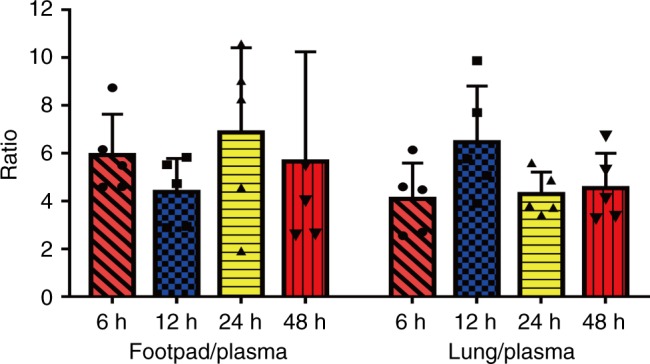
The ratios of footpad/plasma and lung/plasma of TB47 concentrations. BALB/c mice were given a dose of 10 mg kg^−1^ TB47 by oral gavage. Data are expressed as mean ± SD of five samples

Pharmacokinetic parameters of TB47 in BALB/c mice are summarized in Table 2. After intravenous administration of 2 mg kg^−1^ TB47, the AUC (0–*t*) and *t*_1/2_ were 10,409 ± 139 μg L^−1^ × *h* and 17.7 ± 2.6 h, respectively. Whereas the AUC (0–*t*), *C*_max_ and *t*_1/2_ were 19,823 ± 1665 μg L^−1^ × *h*, 0.63 ± 0.28 µg mL^−1^ and 35.6 ± 2.7 h, respectively, after oral administration of 10 mg kg^−1^ TB47, and the bioavailability was 38.1%. The long half-life and high blood concentration of TB47 are likely significant contributors to its potent in vivo activity against *M. ulcerans*. Furthermore, the concentrations in mice foot tissue or lungs at 6, 12, 24, and 48 h were > 4 times higher than in the plasma (Fig. 6).

**Table 2:** Results of pharmacokinetic parameters of TB47 in BALB/c mice

Drug delivering	Parameters	Unit	Mean	SD
**Intravenous injection**	AUC_(0-*t*)_	μg L^−1^ × h	10,409	139
	AUC_(0-∞)_	μg L^−1 ^× h	11,951	565
	MRT_(0-*t*)_	h	14.7	1.7
	MRT_(0-∞)_	h	22.3	3.7
	*t*_1/2z_	h	17.7	2.6
	CL	L h^−1^ kg^−1^	0.17	0.01
	*V*	L kg^−1^	4.27	0.44
	*C*_2min_	μg L^−1^	3709	1805
**Oral administration**	AUC_(0-*t*)_	μg L^−1^ × h	19,823	1665
	AUC_(0-∞)_	μg L^−1 ^× h	33,144	5164
	MRT_(0-*t*)_	h	20.2	0.3
	MRT_(0-∞)_	h	52.0	4.2
	*t*_1/2z_	h	35.6	2.7
	*T*_max_	h	3.2	2.8
	CL	L h^−1^ kg^−1^	0.30	0.02
	*V*	L kg^−1^	15.49	0.38
	*C*_max_	μg L^−1^	626	282

## Discussion

Despite the the efficacy of the standard regimen of streptomycin and rifampin for 2 months for treatment of BU, it has significant disadvantages including daily parenteral administration and potential risk of side effects, such as hearing loss^4,5^. An all-oral and less toxic treatment regimen of Buruli ulcer has been sought after and encouraged by WHO. However, most of newly developed treatments were based on rifampin although it is well known that rifampin causes interactions with many drugs, including anti-retroviral agents and rifampin-resistance in *M. ulcerans* isolates from patients and infected animals have been reported^7,13^. New powerful drugs, especially those with new mechanisms of action could address the current need for novel regimens to treat BU.

TB47 exhibits potent bactericidal activity with very low MIC and MBC against *M. ulcerans*. Detection of MICs of TB47 against *M. ulcerans* on agar plates was repeated only once (duplicate but not triplicate), which may hinder potential reproducibility. However, We (1) used two strains (*M. ulcerans* 1059 and *M. ulcerans* 1615) and they showed the same MICs of TB47 and the MIC detection was duplicated for each strain; (2) repeated the liquid MIC_lux_ detection more than four times by different persons; (3) repeated more than ten times to select TB47-resistant *M. ulcerans* mutants on agar containing as low as 0.05 to 0.1 µg mL^−1^ TB47 but failed. This observation further validates the very low MIC observed for TB47;(4) repeated the MBC determination three times (independent repeats) and confirmed it too is very low. Moreover, it is striking that even at 0.8 mg kg^−1^ of TB47, once daily, it exhibited bactericidal activity and overall superior efficacy than the currently used standard regimen containing rifampin and streptomycin. While mice treated with the standard regimen sustained clinical indications such as swelling of the footpad, such indications were nonexistent in the footpads of mice that received TB47 at doses of ≥ 3.1 mg kg^−1^. In addition, in comparison to the standard regimen, TB47 prolonged the time-to-relapse. This indicated that TB47 has a quick therapeutic effect and could possibly shorten the duration of the BU treatment. Although TB47 alone showed very good activity against *M. ulcerans*, it did not produce 100% cure at 25 mg kg^−1^ daily, 5 days per week, for 2 weeks.

We demonstrate that TB47 targets mycobacterial QcrB because QcrB mutations cause TB47 resistance and deletion of cytochrome bd oxidase (Cyt-bds) makes mycobacteria more susceptible to TB47 (Fig. 4). The differences in Cyt-bds from different bacteria potentially explain their differential susceptibilities to TB47. The *M. smegmatis ΔcydAB* can be a good system to test the function of *cydAB* introduced from other mycobacteria and could be used as a model for screening inhibitors of Cyt-bds. It is valuable to test activity of Cyt-bds in different mycobacteria, such as *M. leprae*. There are no significant similar CydAB proteins in *M. leprae*, which indicated that Cyt-bc1:aa3 could be the only terminal oxidase in the electron transport chain in this mycobacterium. So it is very attractive to test TB47 against leprosy caused by *M. leprae* as it is still a public health concern in many countries despite sustained efforts to eliminate it over the last 30 years. Depending upon the leprosy patient’s disease presentation, they are currently treated for 6, 12, or 24 months with a combination of rifampin, dapsone and with or without clofazimine^14^. Many patients experience side effects, toxicity, and potential drug resistance^15,16^. Our study also supports that the combination of the potential Cyt-bds inhibitors and TB47 may shorten the duration of further for both drug-susceptible and drug-resistant TB. None of atomic structures of mycobacterial bd oxidases has been reported yet but that from *Geobacillus thermodenitrificans* was resolved recently^17^. The structures of mycobacterial CydAB complexes and the rational design of inhibitors of them are highly needed in the discovery and development of antimycobacterial drugs.

In summary, our study demonstrates that TB47 acts by a different mechanism compared to drugs considered to treat BU thereby reducing the chances of development of cross-resistance. In addition, the rate of spontaneous resistance of *M. ulcerans* to TB47 is very low. Oral bioavailability, relatively long half-life and lack of toxicity are additional appealing features of TB47. To leverage these clinically desirable attributes, we propose that a multi-drug combination regimen containing TB47 has promise to produce a stable cure while also potentially shortening the duration of treatment. Further preclinical assessments of dose, frequency and duration of administration would be necessary to harness the optimal potential of TB47 against BU.

## Methods

### Drugs formulation

TB47 was synthesized in a batch and supplied by Guangzhou Eggbio Co. Ltd with purity 98.67% by High Performance Liquid Chromatograph (Batch number: TB47160616). It was formulated in 0.05% CMC-Na (sodium salt of carboxy methyl cellulose) for in vivo study. Rifampin and streptomycin were both bought from TCI (Japan) and dissolved in distilled water for in vivo use. Streptomycin and isoniazid (Macklin, Shanghai) were dissolved in distilled water, and rifampin and TB47 were dissolved in dimethyl sulfoxide (DMSO) for in vitro use. All drugs prepared weekly for in vivo studies. Hygromycin was bought from Roche (Swiss) in liquid form and kanamycin was bought from Sigma (USA). They were dissolved in distilled water for in vitro study.

### Strains and growth conditions

*M. ulcerans* strains 1059 and 1615, *M. marinum*, *M. smegmatis* C^2^155, and derivative strains were grown in Middlebrook 7H9 broth medium with 10% 2-oxo-acid dehydrogenase complexes (OADC) + 0.2% glycerol + 0.05% tween80 for culture and without tween80 for drug susceptibility testing or on 7H11 plates supplemented with 0.2% glycerol + 10% OADC. *M. ulcerans* and *M. marinum* strains were grown at 30 to 32 °C and others at 37 °C. Other clinical important bacteria were grown according to the Clinical and Laboratory Standard Institute Guidelines.

### RLU-based MIC_lux_ and CFU-based MBC determination

Serial dilutions of drug-containing solutions and autoluminescent mycobacteria broth culture (OD_600_ of 0.3 to 1.0) were prepared^6^. RLU counts from the same batch of triplicate samples were measured according to the designed time points. Ninety-six-well plates were measured as rendering the amount of light by using a Veritas™ Microplate Luminometer Operating Manual (Turner BioSystems). MIC_lux_ was determined as the lowest concentration that can inhibit > 90% RLUs compared with that from the untreated controls^6,18^. The time-killing curves and MIC_lux_ of autoluminescent strains were determined by detecting RLUs from these samples. For *M. ulcerans* and *M. marinum* strains, the incubation time for MIC_lux_ reading was 7 days and 3 days, respectively. The minimum bactericidal concentration (MBC) was defined as the lowest concentration of drug that killed ≥ 99% of the CFU compared with the CFU count of the initial inoculum used for the liquid activity assays, which was adopted from reference^19^. In our case, the liquid media in the culture tubes that showed the RLU at least 1 log_10_ lower than that at day 0 were diluted and plated on 7H11 agar (supplemented with 10% OADC) plates for CFU counts. The concentrations used were twofold series of dilutions for this purpose. The incubation time for RLU reading was 14 days for the MBD experiment and the light from each 1.5 mL tube containing total 500 μL culture was detected using Promega GloMax 20/20n. Triplicate tubes for each concentration were plated for CFU counts at d0, 3, and 7 and to detect the quick bactericidal activity of TB47 and to show the correlation of RLU and CFU counts. The concentrations used were fivefold series of dilutions for this purpose. Each above experiment was performed in triplicate (three independent experiments) or more by independent persons and from three or more independent cultures. The representative results are shown.

### MIC determination

The serial tenfold diluted mid-log-phase cultures were plated on 7H11 plates containing different concentrations of drugs. The MICs of TB47 to *M. ulcerans* strains 1059 and 1615 were defined as the lowest concentration that can inhibit at least 99% growth observed from drug-free control plates^7^_._ The experiments detecting such MICs using agar plates were only repeated once (in duplicate) and each time with two different strains. The results were shown as indicated in Supplementary Table 1. MICs against a range of clinically important bacteria were tested using standard broth dilution assay as per the Clinical and Laboratory Standard Institute Guidelines.

### Animal studies

All animal procedures were conducted according to relevant national and international guidelines. All animal care and experimental protocols were approved by the Committee on Laboratory Animal Ethics of Guangzhou Institutes of Biomedicine and Health (GIBH), Chinese Academy of Sciences, #2017077. Serial, non-invasive, real-time monitoring of drug activity in a murine model of Buruli ulcer was used. Colony suspensions of autoluminescent *M. ulcerans* 1059 were made by vortex using 30 mg of fresh colonies in 10 mL PBS and the resulting suspension was used to inject the left hind footpads of six-week-old, female BALB/c mice. The inoculum volume was 0.05 mL, containing approximately 5 to 6 log_10_CFU. The right hind footpads served as negative controls for observation of swelling. The lesion index was defined as follows: index 0 = normal footpad; index 1 = noninflammatory footpad swelling; index 2 = inflammatory footpad swelling; index 3 = inflammatory hind foot swelling^10^. The schemes for testing TB47 activity alone and in combination were demonstrated in Supplementary Table 3 and Supplementary Table 4, respectively. Subcutaneous route for streptomycin and oral gavage for others were used.

### MBD detection

The MBD is defined as the lowest dose able to reduce the footpad CFU counts by 99% compared to the counts in the controls pretreatment, adopted from references^20,21^. Mice were treated for 5 days and sacrificed at d7 as demonstrated in Supplementary Table 3. This experiment was carried out more than triplicate by detecting footpad suspension and only footpad tissue from two independent experiments were plated for CFU counts at d0 and d7 for MBD detection. The representative results are shown in Fig. 1c.

### Selection of spontaneous-resistant mutants to TB47

Broth cultures (OD_600_ from 0.6 to 1.3) of autoluminescent *M. ulcerans* 1059, wild-type *M. ulcerans* 1615, *M. ulcerans* 1059, and autoluminescent *M. marinum* were plated on 7H11 plates containing 0.02, 0.05, 0.1, 0.5, 1, 2, 10, or 40 μg mL^−1^ TB47. The colonies grown upon the TB47-containing plates were picked up to confirm the drug resistance phenotype by real-time in vitro drug susceptibility testing in liquid media or agar method as described above. We repeated about ten times using *M. ulcerans* and each time we plated at least 20 plates (0.5 mL per plate, so > 10 mL culture per passage) at concentrations ≥ 0.1 μg mL^−1^ but three times only at 0.02 and 0.05 μg mL^−1^. And we tried different *M. ulcerans* strains and with tenfold concentrated broth culture. The light from AlMu broth reached >10 million RLU mL^−1^, which indicating more than 10^8^ CFU mL^−1^. The series of tenfold diluted culture was diluted and plated on drug-free plates for detecting the bacterium density.

### Whole-genome sequencing

The genome DNAs of the parent *M. marinum* and the confirmed TB47-resistant mutants were whole-genome sequenced by Beijing Genomics Institute, China. The resulting reads were aligned to the *M. marinum* genome sequence and compared with that of the parent strain.

### Over-expression of *qcrB* genes in *M. marinum* and *M.ulcerans*

Three types (*qcrB*^wild type^, *qcrB*^Thr323Pro^, and *qcrB*^Thr323Ala^) of *qcrB* genes were amplified from genome DNAs of wild-type and spontaneous-resistant *M. marinum* mutants by PCR using primers qcrBmrEf- qcrBmrEr (Table S5) and inserted into the p60luxn plasmid under the control of the *hsp60* promoter. The three plasmids were transformed into the wild-type autoluminescent *M. marinum* or *M. ulcerans*. The MICs_lux_ of the recombinant strains to TB47 were determined as described above.

### Pharmacokinetics

BALB/c mice were given 2 mg kg^−1^ TB47 intravenously or 10 mg kg^−1^ orally. Blood samples were taken from the eyeballs of five mice per time point at 2, 30 min, 1, 2, 6, 12, 24, and 48 h post-dose for intravenous group or at 15, 30 min, 1, 2, 6, 12, 24, and 48 h for oral group. Footpads and lung tissues were collected at 6, 12, 24, and 48 h post-dose for oral group. After extraction and centrifuge at 4 °C, TB47 concentrations were determined by Liquid Chromatograph-Mass Spectrometer (LC-MS).

### Construction of the *M. smegmatis cydAB* knockout mutant

The recombineering method was used^22,23^. The DNA fragments of upstream of *cydA* and downstream of *cydB* of *M. smegmatis* were amplified using primers cydAf-cydAr and cydBf-cydBr (Supplementary Table 5) and cloned into a plasmid by three fragment ligation and verified by sequencing. Then the fragment *dif-Hyg-dif* was inserted in between the two fragments and the resulting *ArmLcydA–dif-Hyg-dif–ArmRcydB* was excised from the plasmid and transformed into induced *M. smegmatis*-TS53 (*M. smegmatis* containing pJV53Ts) competent cells^22^. The *cydAB* genes were replaced by the *Hyg* gene through allelic replacement. To remove the *Hyg* gene, the mutants were cultured in 7H9 broth without hygromycin for 3 days. To remove vector pJV53Ts, the mutants were cultured in 7H9 broth at 42 °C for 3 days, serially tenfold diluted and plated onto 7H11 plates containing 10% sucrose, and incubated at 42 °C for 72 h. The loss of the vector pJV53Ts in the mutants was subsequently confirmed by plating 100 colonies in 7H11 plates containing 10% sucrose and kanamycin or in 7H11 plates containing 10% sucrose at 42 °C. The *M. smegmatis∆cydAB* mutant (Msm*∆cydAB*) was verified by PCR similar to the published design^22^ using primers cyda-d (Supplementary Table 5) and sequencing.

### *CydAB* complementation and growth curves comparison

Complementation plasmids were created by amplifying the *cydAB* genes from genome DNAs of *M. tuberculosis* H37Rv, *M. ulcerans, M. marinum*, and *M. smegmatis* by PCR using primers indicated in Supplementary Table 5 and inserting them into the p60luxn plasmid under the control of the *hsp60* promoter, resulting in the series of plasmids p60luxn*–cydAB*. A series of recombinant *M. smegmatis* strains were constructed as: Msm*∆cydAB*::*cydAB* from *M. tuberculosis*, Msm*∆cydAB*::*cydAB* from *M. ulcerans*, Msm*∆cydAB*::*cydAB* from *M. marinum*, Msm*∆cydAB*:: *cydAB* from *M. smegmatis* and Msm*∆cydAB*:: empty vector p60luxn. Log-phase (OD_600_ = 0.5–0.7) recombinant *M. smegmatis* cultures grown from single colonies were ten thousand-fold diluted and OD_600_ values of the wild-type and recombinant *M. smegmatis* strains were monitored simultaneously at about 4 h intervals till the OD_600_ reached plateau.

### Drug susceptibility comparison of *M. smegmatis* strains

The MICs for wild-type and recombinant *M. smegmatis* strains were determined by using the microplate Alamar blue assay^8^. The log-phase cultures (OD_600_ = 0.6–0.8) were diluted to OD_600_ = 0.001 (~10^5^ CFU mL^−1^). Antibiotics were dissolved in 0.2 mL of diluted culture in the 96-well microplates. Then 20 μL of 10 × alamar blue solution (Alamar Biosciences/Accumed, Westlake, Ohio) was added into each well starting from 36 h after incubation of the plates at 37 °C. Fluorescence units (FU) were recorded 4 h for *M. smegmatis* post-alamar blue addition by using the Envision Multilabel Plate Reader (Perkin-Elmer, Massachusetts, MA, USA) with excitation at 530 nm and emission at 590 nm. The MIC was defined as the lowest concentration that can inhibit > 90% growth. Different *M. smegmatis* strains were cultured until OD_600_ reached ~1.0, then 10 μL of each strain were plated on the same plates containing different concentrations of TB47 and incubated at 37 °C for 36 h.

### Statistical analysis

Experiments were done from at least three biological repeats when possible. RLU and CFU counts were log_10_ transformed before analysis and were presented as mean ± SD. Group means were compared by unpaired Student’s *t*-tests. The significance level was set at *P* < 0.05. Time-to-swelling curves were calculated using the log-rank test. Correlation analysis was performed using Pearson’s correlation test. All statistical tests were performed with Graphpad Prism 7 software.

### Reporting summary

Further information on experimental design is available in the Nature Research Reporting Summary linked to this article.

## Supplementary information

Supplementary Information

Reporting Summary

## Data Availability

Sequence data that support the findings of this study has been deposited in GenBank with accession codes QcrBs (*M. tuberculosis*: AJF03548.1; *M. smegmatis*: AFP40620.1; *M. marinum*: ACC41667.1; *M. ulcerans*: ABL05699.1) CydAs (*M. tuberculosis*: CCP44387.1; *M. smegmatis*: AAF06811.1; *M. marinum*: ACC40876.1; *M. ulcerans*: BAV41839.1) CydBs (*M. tuberculosis*: CCP44386.1; *M. smegmatis*: AAF06812.2; *M. marinum*: ACC40875.1; *M. ulcerans*: BAV41840.1), respectively. The authors declare that all other relevant data supporting the findings of this study are available within the article and its supplementary information files and from the corresponding author upon reasonable request.
