# Supplementary material for: The compound TB47 is highly bactericidal against Mycobacterium ulcerans in a Buruli ulcer mouse model
Source: Nat Commun. 2019 Jan 31;10:524. doi: 10.1038/s41467-019-08464-y (PMC6355801; doi:10.1038/s41467-019-08464-y)
Supplement: Supplementary file 1 — Supplementary Information [file 41467_2019_8464_MOESM1_ESM.docx]

**Supplementary materials:**

**The compound TB47 is highly bactericidal against *Mycobacterium ulcerans* in a Buruli ulcer mouse model**

Liu *et al*.

Supplementary Figures, 1 to 4

Supplementary Tables, 1 to 5


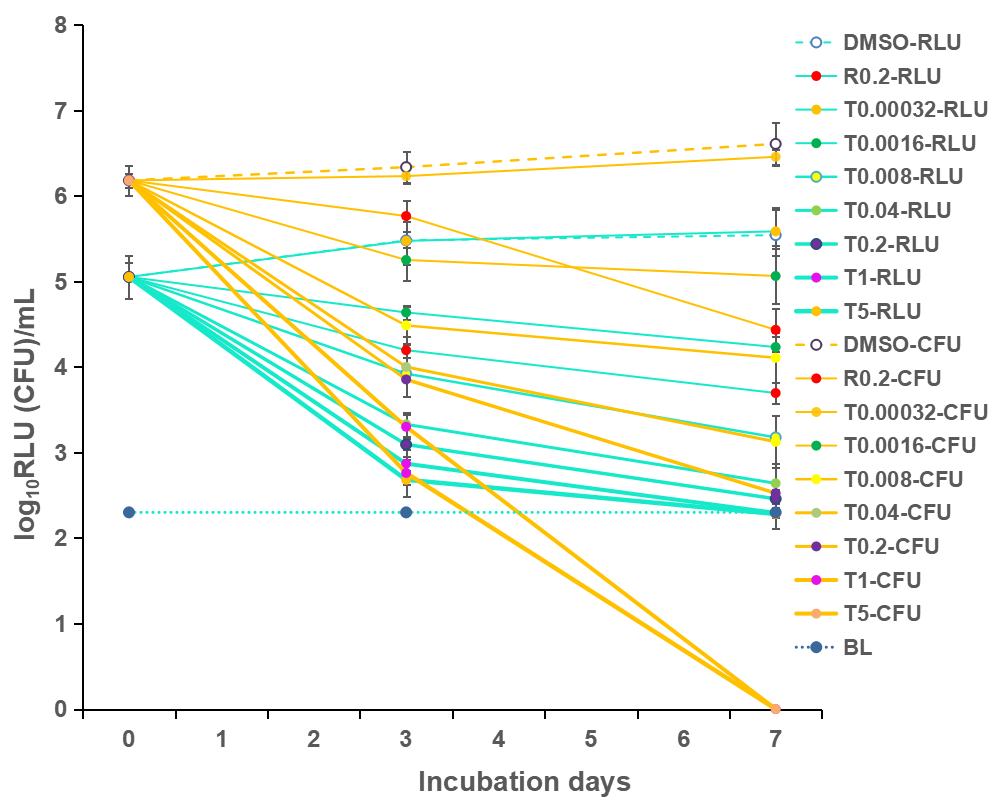


**Supplementary Fig. 1. RLUs and CFUs of *M. ulcerans* culture at day 0, 3 and 7.** T, TB47; BL, base line; Ut, untreated; R, rifampin. Data are expressed as mean ± s.d from three independent biological repeats*.* The correlation coefficients (R^2^) of RLU and CFU for the this experiment were 0.977 (including the positive and negative groups) and 0.985 (TB47 treated groups only), respectively. Correlation analysis was performed using Pearson’s correlation test. The linearity of the relationship of RLU and CFU in broth is valid when the CFUs are from 2.4 to 6.2 log_10_ CFU mL^-1^. The experiment was performed in triplicate (three independent experiments) and the representative results are shown.


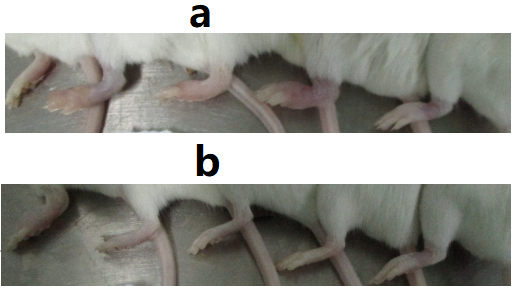


**Supplementary Fig. 2. Photos of mouse footpads from the first animal experiment.** Footpads of mice treated with TB47 25 mg kg^-1^ (a, 3 mice relapsed with one questionable) and TB47 50 mg kg^-1^ (b, 1 mouse relapsed) daily for 5 days and at 24 weeks after treatment completion. 5 mice group^-1^.


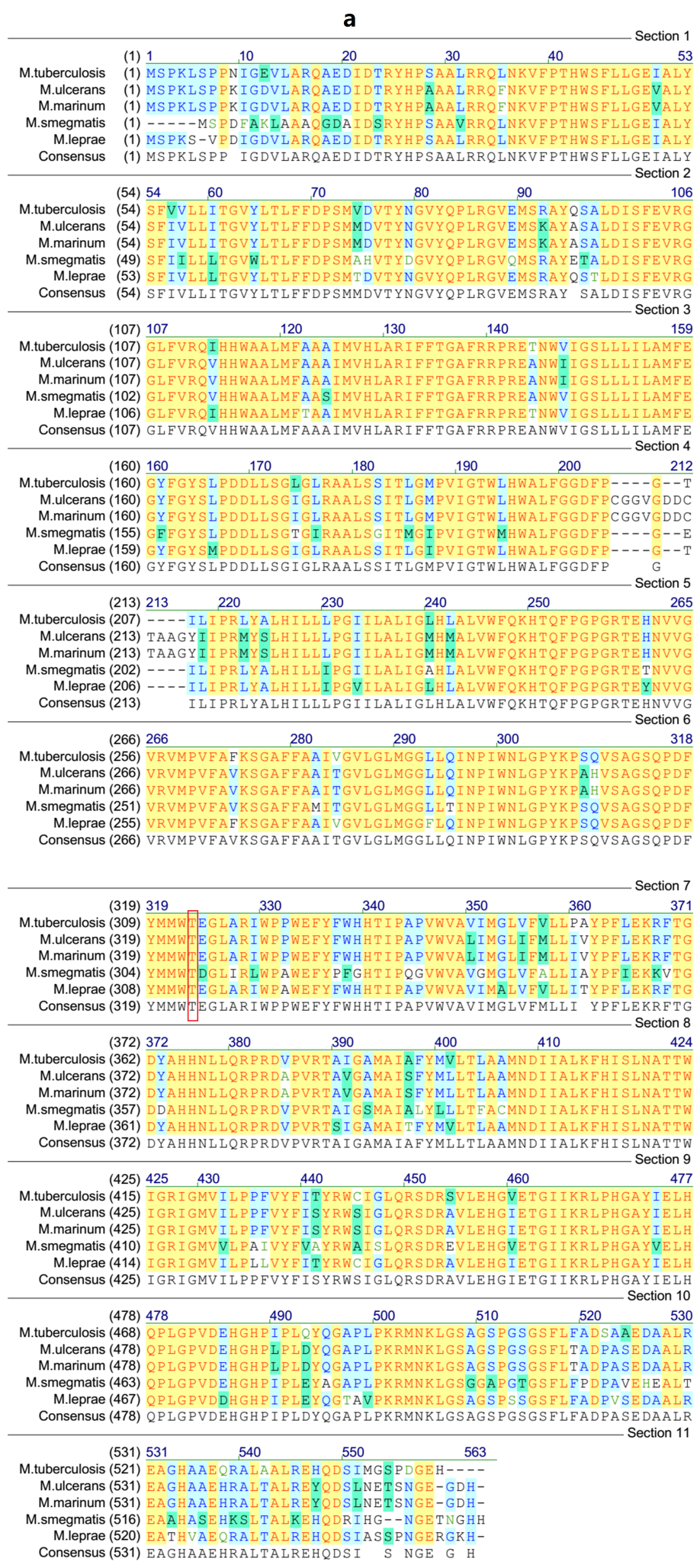


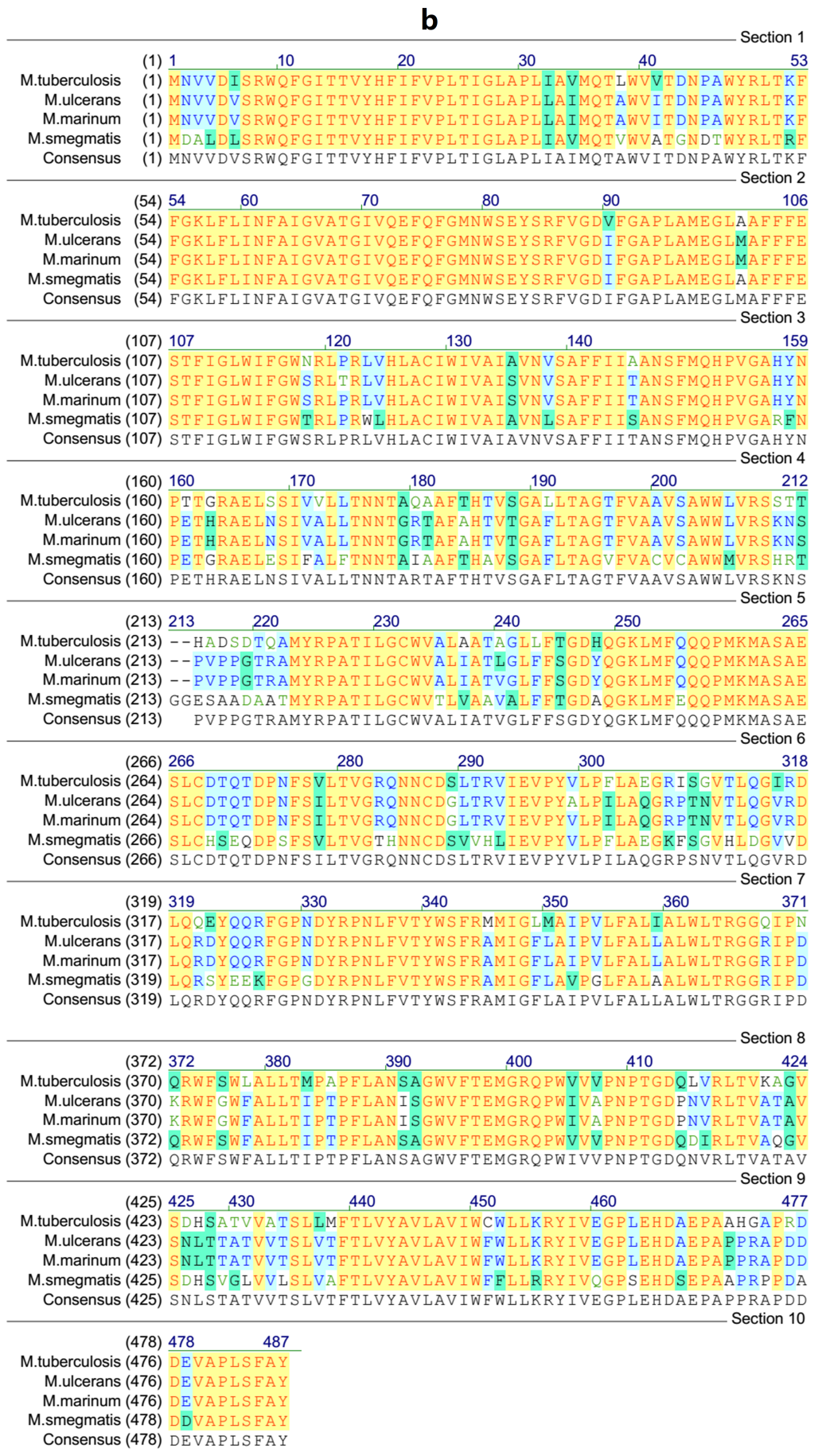


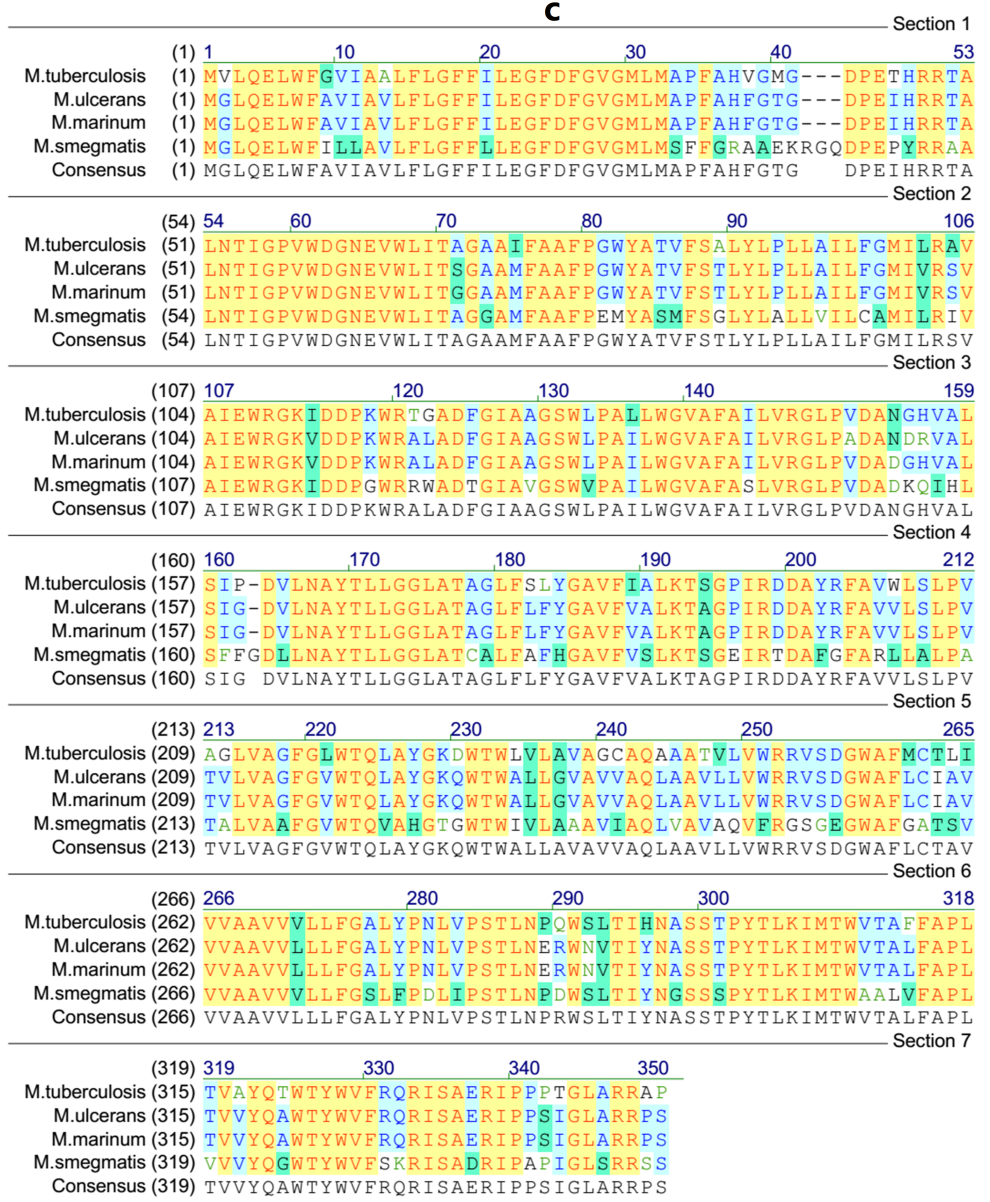


**Supplementary Fig. 3. The amino acid sequences alignments.** **(a)** **QcrB** (The mutation site is indicated in red box. QcrB proteins from *M. ulcerans* and *M. marinum* are identical.), **(b) CydA** , **(c)** **CydB**.


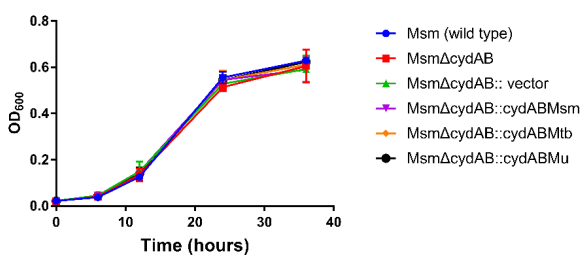


**Supplementary Fig. 4. Growth curves for *M. smegmatis* mc^2^ 155 and derivative strains.** The *cydAB* genes were deleted in *M. smegmatis* mc^2^ 155 leading to strain *M. smegmatis ΔcydAB.* Recombinant *M. smegmatis ΔcydAB*::*cydAB^Msm^*, *M. smegmatis ΔcydAB*::*cydAB^Mtb^, M. smegmatis ΔcydAB*::*cydAB^Mu^* were created by introducing the *cydAB* genes from *M. smegmatis*, *M. tuberculosis* H37Rv and *M. ulcerans*, respectively.

**Supplementary Table 1. Activity of TB47 against different bacteria.**

| **Bacteria** | **Strain No.** | **MIC (μg ml^-1^)** |
| --- | --- | --- |
| *Mycobacterium bovis* BCG Tice | 1 | 0.003 |
| *Mycobacterium ulcerans* | 2 | 0.0016 |
| *Mycobacterium marinum* | 1 | 0.001-0.008 |
| *Mycobacterium smegmatis* C^2^ 155 | 1 | 50 |
| *Mycobacterium abscessus** | 20 | >128 |
| *Klebsiella pneumoniae** | 3 | >128 |
| *Acinetobacter baumannii** | 3 | >128 |
| *Staphylococcus aureus** | 1 | >128 |
| *Escherichia coli** | 5 | >128 |
| *Pseudomonas aeruginosa** | 3 | >128 |
| *Enterococcus faecium** | 1 | >128 |
| *Enterococcus faecalis** | 1 | >128 |
| *Salmonella enteriditis** | 1 | >128 |
| *Candida albicans** | 2 | >128 |

*Clinical isolates identified by 16RNA sequencing.

**Supplementary Table 2. Genotoxicity, potential drug-drug interaction, metabolic stability and [permeability](http://www.baidu.com/link?url=CjhWqZsNwJlEBdZBtYO1fLdUuGyfy92U0qW1kK5pmzV9U2BHpBB02evnIeTD40sotbd3koWXI4i9qL26r66iXfBBUqHkL5fObJjxFxwadUm&wd=&eqid=a0742c950000c9c7000000065bae3c71) of TB47.**

**Table 2-1 Genotoxicity**

| **GreenScreen HC** | | | | **GreenScreen HC + S9** | | | |
| --- | --- | --- | --- | --- | --- | --- | --- |
| **Cytotoxicity** | | **Genotoxicity** | | **Cytotoxicity** | | **Genotoxicity** | |
| Result | LEC | Result | LEC | Result | LEC | Result | LEC |
| Negative | - | Negative | - | Negative | 500 μM | Negative | - |

LEC: The lowest effective concentration. Comment: no Genotoxicity.

**Table 2-2 Drug-drug interaction**

| **CYP Inhibition(IC_50_)** | **1A2** | > 10 μM  (Furaffyline: 6.26 μM) |
| --- | --- | --- |
|  | **2C19** | > 10 μM  (Tranylcypromine: 2.69 μM) |
|  | **2C9** | > 10 μM  (Sulfaphenazole: 0.147 μM) |
|  | **2D6** | > 10 μM  (Quinidine: 0.0777 μM) |
|  | **3A4-Midazolam** | > 10 μM  (Ketoconazole: 0.0390 μM) |
|  | **3A4-Testerone** | > 10 μM  (Ketoconazole: 0.0225 μM) |

**Table 2-3 Metabolic stability**

| Species | T_1/2_ (min) | Clint *In Vitro* (mL min^-1^ gprot^-1^) | Clint *In Vivo* Extpl (mL min^-1^) | Clint Hep *In Vivo*  Extpl (mL min^-1^) | Stability |
| --- | --- | --- | --- | --- | --- |
| Human | Stable | 0.0 | 0.0 | 0.0 | High |
| Rat | 128 | 16.5 | 7.4 | 5.4 | High |
| Mouse | 127 | 16.5 | 1.1 | 0.8 | High |
| Monkey | 93302 | 0.0 | 0.1 | 0.1 | High |
| Dog | 598 | 3.5 | 19.0 | 17.1 | High |

**Table 2-4 [Permeability](http://www.baidu.com/link?url=CjhWqZsNwJlEBdZBtYO1fLdUuGyfy92U0qW1kK5pmzV9U2BHpBB02evnIeTD40sotbd3koWXI4i9qL26r66iXfBBUqHkL5fObJjxFxwadUm&wd=&eqid=a0742c950000c9c7000000065bae3c71)**

| **Flow Direction** | **Mean Recovery %** | **Mean Papp (10^-6^cm s^-1^)** | **Efflux Ratio** | **Conclusion** |
| --- | --- | --- | --- | --- |
| Apical to Basal | 86 | 21.35 | 1.07 | High permeability.  Not P-gp substrate. |
| Basal to Apical | 93 | 22.75 |  |  |

**Supplementary Table 3.** Original experimental scheme to test the preliminary activity TB47 in *M. ulcerans*-infected footpads of BALB/c mice.

| Drug (mg kg^-1^) | Contents/Number of mice to be sacrificed for CFU counts or for live and noninvasive detection^*^  or for relapse in brackets () at the following time points | | | | | | | |
| --- | --- | --- | --- | --- | --- | --- | --- | --- |
|  | CFU on the day  after infection | Treatment initiation,  RLU-L,  RLU-S,  CFU | RLU-L | RLU-L | Treatment  completion | RLU-L | RLU-L,  RLU-S,  CFU,  relapse | Total number of  mice to be sacrificed |
|  | D-11 | D0 | D1 | D3 | D4 | D5 | D7 |  |
| Uninfected |  | 5^*^ | 5^*^ | 5^*^ |  | 5^*^ | 5^*^ | 5^*^ |
| Untreated | 5 | 5,5^*^ | 5^*^ | 5^*^ |  | 5^*^ | 5^*^ | 10,5^*^ |
| R10+ S150 |  | 5^*^ | 5^*^ | 5^*^ |  | 5^*^ | 5^*^ (5) | 5^*^ (5^a^) |
| T 0.4 |  | 5^*^ | 5^*^ | 5^*^ |  | 5^*^ | 5^*^ | 5^*^ |
| T 0.8 |  | 5^*^ | 5^*^ | 5^*^ |  | 5^*^ | 5^*^ | 5^*^ |
| T 1.6 |  | 5^*^ | 5^*^ | 5^*^ |  | 5^*^ | 5^*^ | 5^*^ |
| T 3.1 |  | 5^*^ | 5^*^ | 5^*^ |  | 5^*^ | 5^*^ | 5^*^ |
| T 6.25 |  | 5^*^ | 5^*^ | 5^*^ |  | 5^*^ | 5^*^ | 5^*^ |
| T 12.5 |  | 5^*^ | 5^*^ | 5^*^ |  | 5^*^ | 5^*^ (5) | 5^*^ (5^a^) |
| T 25 |  | 5^*^ | 5^*^ | 5^*^ |  | 5^*^ | 5^*^ (5) | 5^*^ (5) |
| T 50 |  | 5^*^ | 5^*^ | 5^*^ |  | 5^*^ | 5^*^ (5) | 5^*^ (5) |
| Total (85) | 5 | 5,55^*^ | 55^*^ | 55^*^ |  | 55^*^ | 55^*^ (20) | 10, 55^*^ (20) |
| D, day. R, rifampin; S, streptomycin; T, TB47. CFU, colony forming units; RLU, relatively light unit; RLU-L, RLUs detected from footpads of live infected mice; RLU-S, RLUs detected from footpad tissue suspension of sacrificed mice.  ^a^Due to animal welfare reasons, mice were sacrificed early when swelling footpads were found in the relapse monitoring.  ^*^The same batch of 5 live mice were detected for the RLU-L from D0 to D7, and then sacrificed for RLU-S and CFU counts. | | | | | | | | |

**Supplementary Table 4**. Original experimental scheme to compare activities of rifampin + streptomycin and TB47 alone in *M. ulcerans*-infected footpads of BALB/c mice.

| Drug regimens (mg kg^-1^) | Contents/Number of mice to be sacrificed for CFU counts or for live and noninvasive detection^*^  or for relapse in brackets () at the following time points. | | | | | | | | |
| --- | --- | --- | --- | --- | --- | --- | --- | --- | --- |
|  | CFU on  the day  after infection | Treatment initiation,  RLU-L,  RLU-S,  CFU | RLU-L | RLU-L | RLU-L,  relapse | RLU-L,  relapse | RLU-L,  relapse | relapse | Total |
|  | D-11 | D0 | D2 | D4 | Wk1 | Wk2 | Wk5 | Wk6 |  |
| Uninfected |  | 5^*^ | 5^*^ | 5^*^ | 5^*^ | 5^*^ |  |  | 5^*^ |
| Untreated | 5 | 5,5^*^ | 5^*^ | 5^*^ | 5^*^ | 5^*^ |  |  | 10,5^*^ |
| R10 + S150 |  | 10^*a^ | 10^*a^ | 10^*a^ | 10^*a^ | 10^*a^ | 10^*a^(12) | (12) | 10^*a^(24) |
| T 25 |  | 10^*a^ | 10^*a^ | 10^*a^ | 10^*a^ (15) | (15) |  |  | 10^*a^(30) |
| Total (74) | 5 | 5, 10^*^，20^*a^ | 10^*^，20^*a^ | 10^*^，20^*a^ | 10^*^，20^*a^ (15) | 10^*^，20^*a^ (15) | 10^*a^, (12) | (12) | 10,10^*^,(54) |
| D, day; Wk, week. R, rifampin; S, streptomycin; T, TB47.  CFU, colony forming units; RLU, relatively light unit; RLU-L, RLUs detected from footpads of live infected mice; RLU-S, RLUs detected from footpad tissue suspension of sacrificed mice.  ^*^The same batch of 5 live mice were detected for the RLU-L from D0 to D7, and then sacrificed for RLU-S.  ^*a^The same batch of 10 live mice were detected for the RLU-L and then included in the relapse evaluation. | | | | | | | | | |

**Supplementary Table 5. Primers used in this study.**

| **Primers** | **Primer sequences (5’-3’)** | **Purpose** |
| --- | --- | --- |
| qcrBmrf | CCCGTGTCACCAATCCCAGTT | To amplify *qcrB* genes from *M. marinum* strains for checking mutation. |
| qcrBmrr | GCGCGACGCCATAGAACCAA |  |
| qcrBmrEf | GGAATTCCATATGATGAGCCCGAAACTCA | To amplify *qcrB* genes from *M. marinum* strains for overexpression. |
| qcrBmrEr | CCCAAGCTTCTAGTGATCGCCCTCG |  |
| cydAf | CGG**GGTACC**ACCTGTGGAAGGCGACGCT | To amplify left and right arms of DNA for knocking out *cydAB* genes in *M. smegmatis*. |
| cydAr | CCC**AAGCTT**GGTAGACCGTGGTGATTCCG |  |
| cydBf | CCC**AAGCTT**TACTGGGTGTTCAGCAAGCG |  |
| cydBr | CCG**GAATTC**TCATGTCGCCGAACACCAGG |  |
| cyda | TCACCATCGGCGCCTCCCGTG | For verifying the selectable marker free *M. smegmatis ∆cydAB* mutant. |
| cydb | GCGCCGCAGCGCACCGGTCGC |  |
| cydc | ACAGCGGTAACGTAAAATCCG |  |
| cydd | CACAGCGGACCTCTATTCACA |  |
| cydaMuf | GATCGATCATATGCAGGCCCGGGAGATGGCAC | To amplify *cydAB* genes from *M. ulcerans* and from *M. marinum* to complement the *M. smegmatis ∆cydAB* mutant. |
| CydbMur | GATCGATCAAGCTTCCGCCGCTGACCGC |  |
| cydaMtbf | GATCGATCCATATGCTCCGGAGATGACAG | To amplify *cydAB* genes from *M. tuberculosis* to complement the *M. smegmatis ∆cydAB* mutant. |
| CydbMtbr | GATCGATCAAGCTTCGGGCCTTGGTGC |  |
| cydaMsmf | GATCGATCCATATGCAGGCTCGAAAGTAGGTCAG | To amplify *cydAB* genes from *M. smegmatis* to complement the *M. smegmatis ∆cydAB* mutant. |
| CydbMsmr | GATCGATCAAGCTTGCCGAGCGCACGCCAGAGGT |  |
