# Supplementary material for: The compound TB47 is highly bactericidal against Mycobacterium ulcerans in a Buruli ulcer mouse model
Source: Nat Commun. 2019 Jan 31;10:524. doi: 10.1038/s41467-019-08464-y (PMC6355801; doi:10.1038/s41467-019-08464-y)
Supplement: Supplementary file 2 — Reporting Summary [file 41467_2019_8464_MOESM2_ESM.pdf]

nature research

Corresponding author(s): NCOMMS-18-10194627-T

Reporting Summary

Nature Research wishes to improve the reproducibility of the work that we publish. This form provides structure for consistency and transparency in reporting. For further information on Nature Research policies, see Authors & Referees and the Editorial Policy Checklist.

Statistical parameters

When statistical analyses are reported, confirm that the following items are present in the relevant location (e.g. figure legend, table legend, main text, or Methods section).

n/a	Confirmed	
☐	☒	The exact sample size (n) for each experimental group/condition, given as a discrete number and unit of measurement
☐	☒	An indication of whether measurements were taken from distinct samples or whether the same sample was measured repeatedly
☐	☒	The statistical test(s) used AND whether they are one- or two-sided<br>*Only common tests should be described solely by name; describe more complex techniques in the Methods section.*
☒	☐	A description of all covariates tested
☐	☒	A description of any assumptions or corrections, such as tests of normality and adjustment for multiple comparisons
☐	☒	A full description of the statistics including central tendency (e.g. means) or other basic estimates (e.g. regression coefficient) AND variation (e.g. standard deviation) or associated estimates of uncertainty (e.g. confidence intervals)
☒	☐	For null hypothesis testing, the test statistic (e.g. F, t, r) with confidence intervals, effect sizes, degrees of freedom and P value noted<br>*Give P values as exact values whenever suitable.*
☒	☐	For Bayesian analysis, information on the choice of priors and Markov chain Monte Carlo settings
☒	☐	For hierarchical and complex designs, identification of the appropriate level for tests and full reporting of outcomes
☐	☒	Estimates of effect sizes (e.g. Cohen's d, Pearson's r), indicating how they were calculated
☐	☒	Clearly defined error bars<br>*State explicitly what error bars represent (e.g. SD, SE, CI)*

Our web collection on statistics for biologists may be useful.

Software and code

Policy information about availability of computer code

Data collection	Microsoft Excel 2010 and Graphpad prism 7.0 were used to collect and calculate the data
Data analysis	Graphpad prism 7.0 for the statistic analysis and Vector NTI Suite 7 for the alignment were used.

For manuscripts utilizing custom algorithms or software that are central to the research but not yet described in published literature, software must be made available to editors/reviewers upon request. We strongly encourage code deposition in a community repository (e.g. GitHub). See the Nature Research guidelines for submitting code & software for further information.

Data

Policy information about availability of data

All manuscripts must include a data availability statement. This statement should provide the following information, where applicable:

- Accession codes, unique identifiers, or web links for publicly available datasets
- A list of figures that have associated raw data
- A description of any restrictions on data availability

Figure1,3a ,5a,6 and supplementary figure 1, 3, 4 have associated raw data which can be provided if needed. The Accession codes of amino acid sequences of QcrB, CydA, CydB in Figure S2 for Mycobacterium tuberculosis: (AJF03548.1, CCP44387.1, CCP44386.1), Mycobacterium smegmatis: (AFP40620.1, AAF06811.1, AAF06812.2) , Mycobacterium marinum: (ACC41667.1, ACC40876.1, ACC40875.1) and Mycobacterium ulcerans: (ABL05699.1, Mu: BAV41839.1, BAV41840.1)

Field-specific reporting

Please select the best fit for your research. If you are not sure, read the appropriate sections before making your selection.

☒ Life sciences ☐ Behavioural & social sciences ☐ Ecological, evolutionary & environmental sciences

For a reference copy of the document with all sections, see nature.com/authors/policies/ReportingSummary-flat.pdf

Life sciences study design

All studies must disclose on these points even when the disclosure is negative.

Sample size	The sample size in the animal experiment was determined by referring the published papers[Antimicrob Agents Chemother. 49(6):2289-93. (2005);PLoS Negl Trop Dis. 7, e2598 (2013), PLoS Negl Trop Dis. 9, e0003823 (2015)] and the space of the animal facility
Data exclusions	No date were excluded.
Replication	All attempts at replication were successful. Some pilot study results were not reported here and they could be reproduced.
Randomization	Animals were block randomized.
Blinding	The founders had no role in study design, data collection and analysis, decision to publish, or preparation of the manuscript. The first report that TB47 was very powerful in vivo against M. ulcerans infection was by author Yamin Gao, who did not know what the compound was when he used it. Then the formal in vivo experiment against M. ulcerans infection was done by Yang Liu and Yamin Gao together.

Reporting for specific materials, systems and methods

Materials & experimental systems

n/a	Involved in the study	
☐	☒	Unique biological materials
☒	☐	Antibodies
☐	☒	Eukaryotic cell lines
☒	☐	Palaeontology
☐	☒	Animals and other organisms
☒	☐	Human research participants

Methods

n/a	Involved in the study	
☒	☐	ChIP-seq
☒	☐	Flow cytometry
☒	☐	MRI-based neuroimaging

Unique biological materials

Policy information about availability of materials

Obtaining unique materials	Autoluminescent Mycobacterium ulcerans 1059, Autoluminescent Mycobacterium marimun, Selectable marker free autoluminescent Mycobacterium tuberculosis H37Rv strains and other clinical bacterial stains.

Eukaryotic cell lines

Policy information about cell lines

Cell line source(s)	VERO and Thp-1 were purchased from the Shanghai Zhong Qiao Xin Zhou Biotechnology Co.,Ltd. shanghai , China. This is very common.
Authentication	No further authentication of the cell lines was performed before use.
Mycoplasma contamination	The cell lines were tested negative for Mycoplasma contamination
Commonly misidentified lines (See ICLAC register)	N/A

Animals and other organisms

Policy information about studies involving animals; ARRIVE guidelines recommended for reporting animal research

Laboratory animals	Six-week-old female or male BALB/c mice were purchased from Vital River Laboratory Animal Technology Co. Ltd, Beijing, China.
Wild animals	The study did not involve wild animals.
Field-collected samples	The study did not involve samples collected from the field.
